# Determination of growth-coupling strategies and their underlying principles

**DOI:** 10.1186/s12859-019-2946-7

**Published:** 2019-08-28

**Authors:** Tobias B. Alter, Birgitta E. Ebert

**Affiliations:** 1 Institute of Applied Microbiology, RWTH Aachen University, Aachen, Germany; 2 Present Address: Australian Institute for Bioengineering and Nanotechnology, The University of Queensland, Brisbane, QLD 4072 Australia

**Keywords:** Growth-coupled production, Bilevel algorithms, Stoichiometric modeling, Model-guided metabolic engineering, Optimality principles

## Abstract

**Background:**

Metabolic coupling of product synthesis and microbial growth is a prominent approach for maximizing production performance. Growth-coupling (GC) also helps stabilizing target production and allows the selection of superior production strains by adaptive laboratory evolution. To support the implementation of growth-coupling strain designs, we seek to identify biologically relevant, metabolic principles that enforce strong growth-coupling on the basis of reaction knockouts.

**Results:**

We adapted an established bilevel programming framework to maximize the minimally guaranteed production rate at a fixed, medium growth rate. Using this revised formulation, we identified various GC intervention strategies for metabolites of the central carbon metabolism, which were examined for GC generating principles under diverse conditions. Curtailing the metabolism to render product formation an essential carbon drain was identified as one major strategy generating strong coupling of metabolic activity and target synthesis. Impeding the balancing of cofactors and protons in the absence of target production was the underlying principle of all other strategies and further increased the GC strength of the aforementioned strategies.

**Conclusion:**

Maximizing the minimally guaranteed production rate at a medium growth rate is an attractive principle for the identification of strain designs that couple growth to target metabolite production. Moreover, it allows for controlling the inevitable compromise between growth coupling strength and the retaining of microbial viability. With regard to the corresponding metabolic principles, generating a dependency between the supply of global metabolic cofactors and product synthesis appears to be advantageous in enforcing strong GC for any metabolite. Deriving such strategies manually, is a hard task, due to which we suggest incorporating computational metabolic network analyses in metabolic engineering projects seeking to determine GC strain designs.

**Electronic supplementary material:**

The online version of this article (10.1186/s12859-019-2946-7) contains supplementary material, which is available to authorized users.

## Background

Metabolic engineering approaches strive to optimize microbial cell-factories for robust, profitable, and sustainable industrial applications [1]. One applied principle within this field of research is to metabolically couple the synthesis of the product of interest to microbial growth by appropriate genetic modifications [2–6]. The main motivation in generating growth-coupled production is to shift the tug of war for the substrate carbon towards the synthesis of the desired chemical [7–9]. Consequently, growth-coupling (GC) efficiently facilitates the use of well-established adaptive laboratory evolution methods for production strain optimization purposes by employing growth as a simple selection criterion [10, 11].

**Fig. 1:**
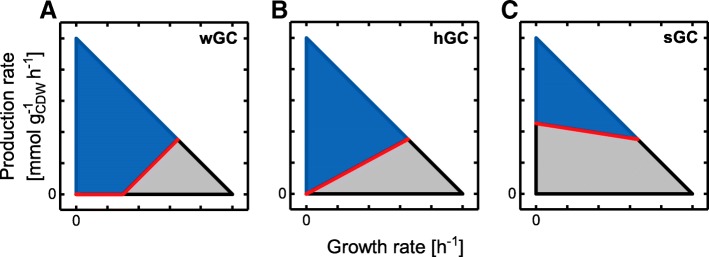
Exemplary production envelopes showing three distinct types of GC. **a** weak, **b** holistic and **c** strong growth-coupling. The grey area represents the production envelope of the wild-type strain which is inaccessible for the mutant strain. The lower production rate bound, hence the minimally guaranteed production rate, is marked by the red line

Three distinct GC phenotypes differing in GC strength can be distinguished, which become apparent from computing and plotting so-called metabolic production envelopes [12]. These production envelopes are projections of the accessible flux space onto the 2D plane spanned by the growth rate and the production rate of the target metabolite (Fig. 1). The lower limit of a production envelope depicts the minimally guaranteed production rate for the accessible range of growth rates. Hence, a lower bound greater than zero for a particular growth state directly implies GC. In the following, production envelopes, in which a production rate greater than zero only occurs at elevated growth rates, will be denoted as a weak GC (wGC) characteristic (Fig. 1a). For *Saccharomyces cerevisiae* and *Escherichia coli*, for example, such a wGC is naturally observed for fermentation products, e.g., ethanol or acetate, under anaerobic conditions or during overflow metabolism. By this means, holistic GC (hGC) is encountered if the lower production rate bound is above zero for all growth rates greater than zero (Fig. 1b) while strong GC (sGC) is referred to production envelopes showing a mandatorily active target compound production for *all* metabolic states including zero growth (Fig. 1c). The physiological equivalent of an sGC behavior in a microbial strain is the concurrent secretion of a metabolite during carbon source consumption independent of the carbon uptake and growth rate, i.e., the metabolite is a necessary byproduct of carbon metabolism. Besides the oxidation byproduct CO_2_, native examples for such mandatory byproduct secretion are, e.g., acetate and lactate formation by acetogenic bacteria during growth on CO_2_ and H_2_ and lactic acid bacteria with obligate homo- or heterofermentative metabolism, respectively. Note that sGC can only be achieved in silico if a positive minimal value for the ATP maintenance requirement (ATPM) reaction is enforced. This constraint precludes the zero flux vector from the solution space and enables the identification of sGC strategies using reaction deletions only [13]. Hence, if not stated otherwise, we employ or refer to models including a minimal constraint on the ATPM reaction in this work.

Various computational algorithms exploiting the rich information content of stoichiometric metabolic models have been developed to specifically provide reaction deletion strategies leading to GC. These approaches are generally grouped into Flux Balance Analysis (FBA) and Elementary Modes Analysis (EMA) based methods. Classical FBA focuses on a particular metabolic phenotype by optimizing a biological meaningful objective function subject to steady-state mass balance constraints [14]. Thus, GC strain designs identified by FBA-based frameworks such as OptKnock [15] and RobusKnock [16] enforce GC at only distinct metabolic states, which is maximal biomass formation in the given examples. While OptKnock attempts to solely maximize the target compound production, RobustKnock maximizes a *minimally guaranteed* production and thus enforces GC at maximal growth. Complementarily to FBA, EMA utilizes the nondecomposable steady-state flux distributions, called elementary modes (EMs), of a metabolic network from which any feasible flux state can be derived by linear combinations of these EMs [17, 18]. By exploiting the nondecomposibility feature of EMs, minimal sets of reaction deletions, coined minimal cut sets (MCSs), can be identified that disable all EMs responsible for undesired metabolic functionalities [19]. Methods such as constrained minimal cut sets [20, 21] or minimal metabolic functionality [4] use EMA to determine MCSs, which remove all EMs producing only biomass and hence lead to GC. The main disadvantage of EMA-based compared to FBA-based methods is the computationally expensive necessity to enumerate all EMs, thus limiting the application of EMA to small or mid-scale metabolic networks. Recently, this has been overcome by introducing MCSEnumerator, an algorithm that sequentially enumerates the smallest MCSs and significantly reduces the computational costs [22]. Still, the underlying constrained MCS method requires the definition of a minimal bound on growth rate and target product yield. Depending on the user-defined boundary conditions, this may result in neglection of the best possible but suboptimal solutions, that is wGC or hGC when no sGC solutions exist for the user-defined maximum allowable number of reaction deletions. To effectively gain from the advantages of different methods in terms of a biologically robust strain design, combinations and adaptions of the mentioned algorithms have been reported [12, 23, 24].

Beside the in silico identification of GC intervention strategies, research on the general feasibility and driving forces of the coupling between growth and target product synthesis has been conducted. Based on the EMA approach, Klamt & Mahadevan [25] have built a theoretical framework to relate GC to the existence of elementary modes and vectors that fulfill specific requirements on biomass and product yields. By applying this framework to a metabolic model of the central carbon metabolism of *E. coli* [4, 20], they were able to show that synthesis of any metabolite can be coupled to growth. Recently, Jouhten et al. [9] proposed a biochemical basis for the generation of growth-coupled product synthesis. They introduced the concept of anchor reactions, which split the substrate carbon among one or more biomass precursors and the target compound. Existence of an anchor reaction that is or can be made essential for the synthesis of a biomass precursor thus implies feasibility of growth-product coupling. This has similarly been expressed by Klamt & Mahadevan [25] in the requirement for at least one elementary mode allowing for both growth and product synthesis. In contrast, it was claimed elsewhere that GC results from an induced imbalance of reduction or energy equivalents, which can only be overcome by active product synthesis [5, 23, 26]. Erdrich et al. [26] pointed out, that this imbalance is particularly pronounced under anaerobic conditions where oxygen as final electron acceptor is missing and ATP generation is naturally limited mainly to fermentation pathways and glycolysis.

In view of these disparate explanations for GC, we aimed at further unraveling key principles of reaction deletion strategies leading to GC by identifying relevant genetic intervention strategies for a set of metabolites and investigating the specific operating principle of these strategies. We adapted the mixed-integer linear program formulation of OptKnock to determine GC knockout strategies for a given target compound, a specific substrate and a defined maximum number of reaction deletions. Particularly, our framework, which we termed gcOpt, maximizes the minimally guaranteed production rate at a medium, fixed growth rate and was applied to calculate GC intervention strategies for a broad range of metabolites of a core as well as a genome-scale metabolic model of *E. coli*. The resulting strategies were subsequently examined regarding the consequence of imposed growth-coupled product synthesis on metabolic network operation.

## Results

### gcOpt prioritizes strain designs with an elevated growth coupling strength

**Fig. 2:**
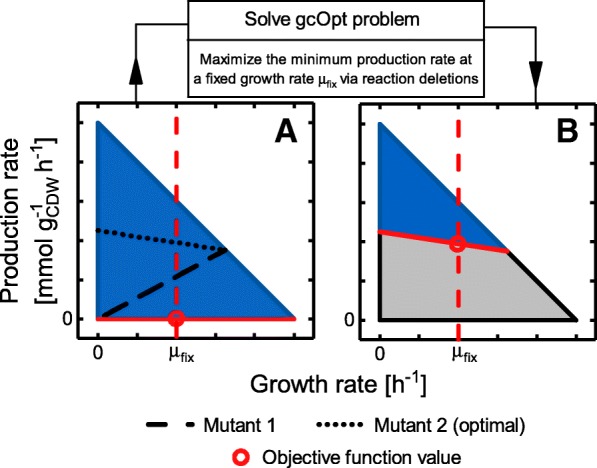
Schematic principle of gcOpt. **a** represents an exemplary production envelope of a wild-type strain showing no GC, with the black dashed and dotted lines denoting the lower production rate bounds of possible mutant strains. The red dashed line denotes the optimization principle of gcOpt, which is maximization of the minimally guaranteed production rate at a medium fixed growth rate. **b** is a production envelope of a reaction deletion mutant strain showing the best possible GC, where the grey area represents the production envelope of the wild-type strain which is inaccessible for the mutant strain

The pursued approach to identify GC strain designs with maximal possible GC strength was derived from the production envelope representation of GC mutants (cf. Fig. 1). While the GC classification into wGC, hGC, and sGC provides a qualitative notion of the GC strength, the position of the lower production rate boundary can be interpreted as a quantitative measure: the higher the boundary in terms of positive rate values, the stronger the GC. The shape of this production envelope boundary along the growth rate axis is not arbitrary. It is rather a part of the hull giving the admissible flux space and, since the flux space is determined by a linear equation system, the lower production envelope boundary is convex [27]. It follows from the convexity property that by increasing the lower production rate for one specific growth rate by, e.g., deletion of one or more reactions, the lower production rate boundary at all admissible growth rates is raised, resulting in an overall increase of the GC strength. This principle was implemented in a bi-level optimization algorithm, gcOpt, which maximizes the minimum production rate of a target compound at a fixed, medium growth rate μ_fix_ using appropriate reaction deletions (Fig. 2). Ultimately, gcOpt provides strain designs with high GC strengths for the production of a specified target metabolite. The theoretically maximal GC strength, however, may be restricted due to the structure of the given metabolic network, the chosen environmental conditions and the defined maximum number of modifications, in which case gcOpt inherently allows for the identification of suboptimal designs (see the Methods section for a detailed description and formulation of gcOpt).

Identification of strain designs leading to GC of ethanol production in *E. coli* under anaerobic conditions was used to demonstrate the functionality of gcOpt. This classic example has already been investigated by applying diverse computational methods to a metabolic model of the central carbon metabolism of *E. coli*, here referred to as CT86 [4, 20]. Using CT86, gcOpt was applied allowing maximum numbers of reaction deletions from one to five at three different fixed growth rates μ_fix_ of 0.01 h^− 1^, 0.1 h^− 1^ and 0.25 h^− 1^. Anaerobic growth on glucose was simulated by setting the maximum glucose and oxygen uptake rate to 12 mmol g^− 1^ h^− 1^ and zero, respectively. The respective reaction deletions of each identified strain design as well as the strategies from literature are given in Additional file 4: Table S1. By applying gcOpt as well as OptKnock, an exhaustive enumeration of GC strain designs from one to five reaction deletions was additionally conducted for the target products succinate and lactate to support the following findings (refer to the Additional file 3: Figures S1 and S2 as well as Additional file 4: Tables S2 and S3 for the corresponding production envelopes and deletion strategies, respectively).

**Fig. 3:**
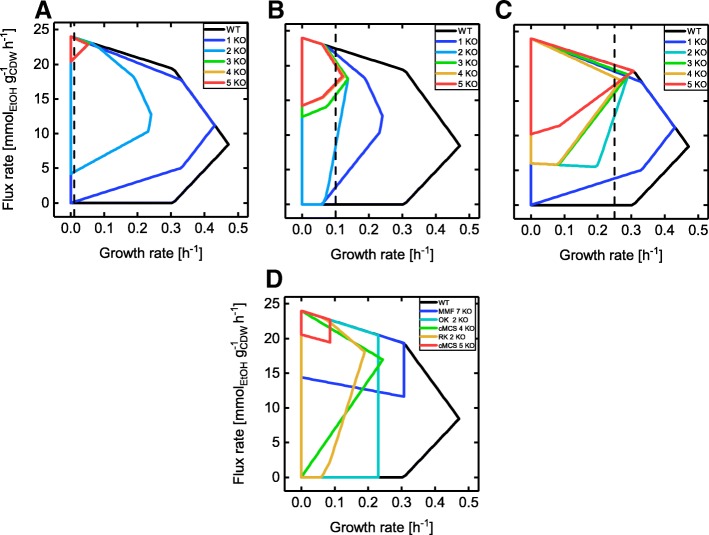
Ethanol production envelopes of GC strain designs identified by gcOpt in comparison to designs taken from literature. Maximal intervention sizes between one and five reaction deletions were used (**a**-**c**) and compared to several methods reported in the literature (**d**) (MMF strategy from [4], all others from [20]). Black lines denote the production envelopes of the wild-type. The vertical black dashed lines mark the chosen fixed growth rates *μ*_*fix*_ for the respective computations (0.01 *h*^−1^ (**a**), 0.1 *h*^−1^ (**b**) and 0.25 *h*^−1^ (**c**)). The maximal glucose uptake rate was constrained to 12 mmol g^−1^ h^−1^ for all respective simulations

The designs identified by gcOpt (Fig. 3a-c) clearly indicate that the lower production rate bound, and hence the GC strength, increased with a growing number of simultaneous reaction deletions while the maximal growth rate decreased and approached the chosen μ_fix_ (refer to Additional file 3: Figure S3 for the corresponding yield spaces). The most extremely trimmed production envelope was computed for the triple, quadruple and quintuple mutants at a μ_fix_ of 0.01 h^− 1^ (Fig. 3a). The maximum growth rates did not exceed values of 0.05 h^− 1^ while an ethanol production rate of approximately 20 mmol g^− 1^ h^− 1^, or a corresponding yield of 1.7 mol mol^− 1^, was strictly guaranteed implicating a tight metabolic coupling of growth and ethanol production. Frequent reaction deletions include the fermentation pathways to prevent the secretion of e.g., lactate and formate, which is in line with GC strain designs given by Trinh et al. [4] and Hädicke et al. [20]. In contrast to these previously reported strategies, the gcOpt designs for a μ_fix_ of 0.01 h^− 1^ and 0.1 h^− 1^ consistently target the upper glycolysis pathway, e.g., the glucose-6-phosphate isomerase or the triosephosphate isomerase. However, the quintuple mutant design computed for μ_fix_ = 0.25 h^− 1^ (Fig. 3c) was interesting in that it enforced a high ethanol production rate at a relatively high maximal growth rate of 0.31 h^− 1^. The minimally guaranteed production rate was 10.2 mmol g^− 1^ h^− 1^ (yield of 0.85 mol mol^− 1^), thus pointing to an excellent combination of GC and viability of this mutant. The predicted intervention strategies at a μ_fix_ = 0.1 h^− 1^ a (Fig. 3b) were a good compromise between this and the extremely trimmed strain designs at a μ_fix_ = 0.01 h^− 1^ with guaranteed production rates of approximately 14.2 mmol g^− 1^ h^− 1^ (yield of 1.2 mol mol^− 1^) and maximal growth rates of 0.13 h^− 1^. Figure 3D contrasts production envelopes of GC strain designs found by various other methods to those identified by gcOpt (Fig. 3a-c). By consulting the lower bounds of the production envelopes as a measure for the GC strength, the double and quadruple mutants determined by OptKnock, RobustKnock and cMCS [20], respectively, generally showed inferior GC characteristics than mutants of the same intervention sizes found by gcOpt. Moreover, although cMCS and the minimal metabolic functionality (MMF) [4] method identified a tight GC for the quintuple and septuple mutants, for both mutant strains the product yield at maximal growth could take a range of values. A bottleneck in biomass precursor supply at elevated growth rates can be assumed in these cases since such edges of flux polyhedra in general, and thus of production envelopes in particular, correspond to flux capacity constraints [25]. Such a phenomenon, however, was not seen for any gcOpt strain design and thus might be avoided by this algorithm.

Consequently, gcOpt offers the advantage to compute attractive GC strain designs for a given microbial host, target compound, environmental condition and specified maximum number of genetic interventions. The inevitable compromise between the predicted viability of GC mutants (the maximal growth rate) and the expected GC strength can furthermore be controlled by adapting the fixed growth rate μ_fix_. Reducing μ_fix_ gradually favors the identification of strain designs with higher GC strengths, thus elevated guaranteed target production rates but possibly lower maximal growth rates. Moreover, the inherent approach of increasing the minimum production rate enforces the generally preferred sGC and hGC solutions, which guarantee product synthesis with growing or metabolically active organisms. This is a beneficial trait compared to alternative FBA based algorithms such as OptKnock or RobustKnock, which per se do not favor these designs over wGC solutions. In terms of computing time, these algorithms are similarly costly as compared to gcOpt due to the same mixed integer linear program (MILP) formulation (cf. Methods section).

### Do metabolic principles leading to growth-coupling exist?

As mentioned in the introduction, there is a diverse discussion about possible principles and routes to enforce GC. These range between pure stoichiometric forces, such as anchor reactions, to flux-based notions which relate GC to imbalances in the households of energy and redox equivalents. As a first computational screening, we applied gcOpt to compute a comprehensive dataset of GC designs, which we analyzed in-depth to decipher general metabolic principles that trigger GC. To this end, we computed intervention strategies with one to seven reaction deletions for the 36 central carbon metabolites of the *E. coli i*AF1260 core model under aerobic as well as anaerobic conditions. The corresponding reaction and gene deletion set of each identified strategy can be found in the Additional files 1 and 2.

Under both anaerobic and aerobic condition, gcOpt simulations were additionally conducted with a decreased as well as an increased non-growth associated maintenance (NGAM) ATP requirement by changing the lower flux bound of the corresponding ATPM reaction (Eq. 1) about 50% from its standard value of 8.39 mmol g^− 1^ h^− 1^ [28] to 4.2 mmol g^− 1^ h^− 1^ and 12.2 mmol g^− 1^ h^− 1^, respectively.

1 $$ ATP+{H}_2O\to ADP+{H}^{+}+ Pi $$

Equivalently to simulating the influence of the NGAM demand on finding GC strain designs, NGAM reactions were separately introduced for NAD(^+^/H) and NADP(^+^/H), virtually resembling an elevated turnover of these cofactors (Eqs. 2–3). Based on metabolic flux analyses of the NADH oxidase gene *nox* overexpression in *Pseudomonas putida* KT2440 [29], the allowable flux range for the consumption of the oxidized or reduced cofactor was set between 5.0 mmol g^− 1^ h^− 1^ and 20.0 mmol g^− 1^ h^− 1^, respectively.

2 $$ NA{D}^{+}+{H}_{in}^{+}\leftrightarrow NA DH $$

3 $$ NA{DP}^{+}+{H}_{in}^{+}\leftrightarrow NA DPH $$

Equations 2 and 3 are mass but not charge balanced to allow for the inclusion of principally any electron donor/acceptor. It is assumed that the electrons are transferred from/to an imaginary electron donor/acceptor, which can be freely reduced or oxidized to avoid mass imbalances of additionally included redox cofactors. In this way the necessity to oxidize /reduce a particular electron acceptor/donor and the corresponding influence on particular metabolic flux routes is circumvented. For each altered ATPM and virtual cofactor NGAM, GC strain designs were successfully identified for approximately 90% of all metabolites under aerobic conditions, except for the condition of increased NADP^+^ consumption, which reduced the coupleable metabolites to 75%. The four metabolites, which could not be coupled to growth, were acetyl-CoA and succinyl-CoA, due to the model’s inability to compensate for the CoA drain, acetyl phosphate, and L-glutamine. For anaerobic growth, the percentage of growth-coupled metabolites was much lower. Interestingly, metabolites, for which gcOpt computed only wGC designs for standard conditions, could be strongly growth-coupled when the ATP NGAM was reduced. Among those were, e.g., phosphorylated intermediates of glycolysis such as glucose-6-phosphate and 2-phosphoglycerate. The growth-coupled synthesis of those metabolites was apparently fueled by excess ATP.

**Fig. 4:**
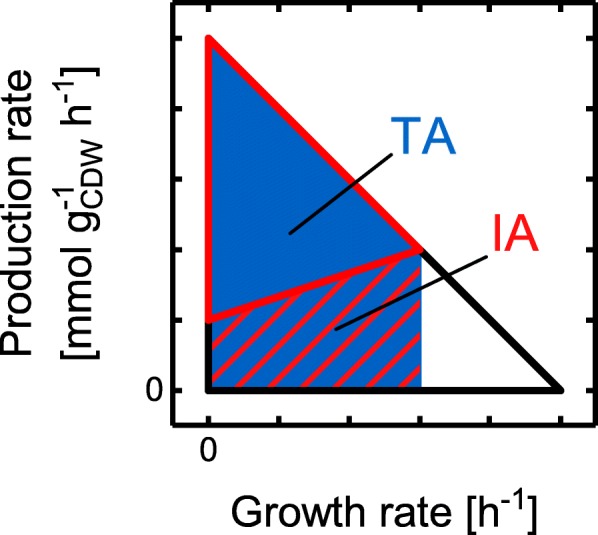
Illustration of the yield space areas used for calculating GCS. Scheme of a wild-type yield space showing no GC (black hull curve) and a GC strain design (red hull curve). The blue area TA illustrates the yield space of the wild-type up to the maximal growth rate of the mutant strain. The inaccessible yield space IA below the lower yield bound of the mutant is marked by the red hatched area

To more quantitatively compare GC characteristics between different designs, a novel measure for the GC strength, termed GCS, was introduced (cf. Methods). GCS relates the area of the accessible production envelope of the wild-type strain to the inaccessible or blocked area below the lower production rate bound of the mutant strain up to the maximum growth rate of the mutant (cf. Figure 4). Thus, the higher the lower production rate boundary of the mutant, the higher the GCS. The minimally guaranteed yield of the target compound at maximal growth of the mutant strain is considered as an additional factor for determining the GCS (Eq. 6) to also incorporate the production capabilities at physiologically relevant growth conditions. Exemplarily and for a better tangibility of the concept, Table 1 shows GCS values for all strategies depicted in Fig. 3.

**Table 1:** Computed growth coupling strength values for all strategies shown in Fig. 3. GCS values of the respective strain designs and number of reaction deletions are colored according to the GC classification of increasing GC strength from wGC (red), hGC (blue) to sGC (green). The values in bold mark the highest GCS among the GC strain designs from literature (D) as well as from gcOpt with a fixed growth rate μ_fix_ of 0.01 h^−1^ (A), 0.1 h^−1^ (B) and 0.25 h^−1^ (C), respectively. The MMF strain design is taken from [4]. All other designs in column D are taken from [20]

μ_fix_ = 0.01 h^–1^ (A)		μ_fix_ = 0.1 h^–1^ (B)		μ_fix_ = 0.25 h^–1^ (C)		Literature (D)	
Design	GCS	Design	GCS	Design	GCS	Design	GCS
WT	-1.97	WT	-1.97	WT	-1.97	WT	-1.97
1 KO	-0.91	1 KO	-1.91	1 KO	-0.91	MMF 7 KO	0.27
2 KO	0.16	2 KO	-1.87	2 KO	0.26	OK 7 KO	No GC
3 KO	**0.63**	3 KO	0.40	3 KO	0.35	cMCS 4 KO	-0.75
4 KO	0.58	4 KO	0.43	4 KO	0.31	RK 2 KO	-1.83
5 KO	0.58	5 KO	**0.45**	5 KO	**0.48**	cMCS 5 KO	**0.52**

**Fig. 5:**
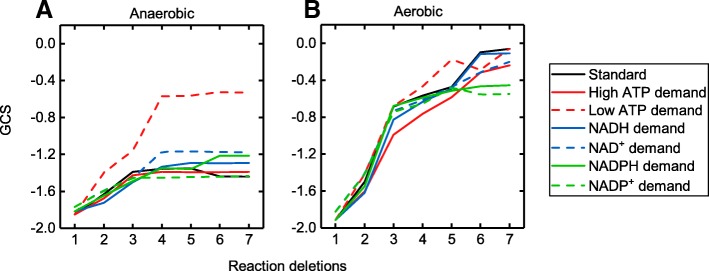
Mean GCS progressions as a function of the number of reaction deletions. GC strain designs were identified by gcOpt for all metabolites of the *E. coli i*AF1260 core model under anaerobic (**a**) and aerobic (**b**) conditions. The different lines embrace independent simulations applying a particular cofactor demand as illustrated by the legend

Figure 5 shows the mean GCS of all investigated metabolites for an increasing number of maximal reaction deletions for anaerobic as well as aerobic conditions and for altered or additionally introduced cofactor requirements. If no GC strain design was identified for a metabolite, the GCS was set to − 2, defined as a complete lack of a coupling between growth and product synthesis. Under anaerobic conditions (Fig. 5a), the mean GCS steadily increased with cumulative reaction knockouts from one to four and reached a plateau above this threshold for all investigated conditions. As already apparent form the increased number of sGC designs (Table 2), a reduced ATP demand, i.e., a low NGAM requirement, increased the mean GCS while alterations of the demand of the redox cofactors NAD(P/H) did not have a comparable effect. For aerobic conditions, we found coupling strategies with significantly higher mean GCS values. 5 B). Again, the mean GCS steadily increased with a growing number of reaction knockouts. The increase attenuated but did not reach a plateau in simulations restricted to maximal seven reaction deletions.

**Table 2:** Percentage of metabolites for which strain designs of type wGC, hGC or sGC were identified. Strain designs were computed by gcOpt with the *E. coli i*AF1260 core model and glucose as the sole carbon and energy source. The total number of investigated carbon metabolites was 36

	Metabolites for which the best GC strategy was of type [%]			Metabolites that could be coupled to growth [%]
	wGC	hGC	sGC	
Aerobic				
standard	6	6	78	89
ATPM high	17	3	69	89
ATPM low	6	11	72	89
NADH demand	17	3	69	89
NAD^+^ demand	17	11	61	89
NADPH demand	33	6	50	89
NADP^+^ demand	25	11	39	75
Anaerobic				
standard	14	3	19	36
ATPM high	3	14	17	33
ATPM low	0	3	53	56
NADH demand	3	14	19	36
NAD^+^ demand	3	6	31	39
NADPH demand	3	14	25	42
NADP^+^ demand	3	8	19	31

### Does product-coupled biomass precursor synthesis exhaust the GC potential?

A possible principle leading to GC, recently discussed by Jouhten et al. [9], is the dependence of the synthesis of one or more biomass precursors on the activity of the target production, e.g., by restricting precursor synthesis to reactions that split the substrate into a precursor essential for growth and the target metabolite. This assumption was tested by applying each found GC design to the *i*AF1260 core metabolic model and computing the capability of the impaired metabolic network to synthesize each reactant of the biomass synthesis equation while disabling the production of the respective target metabolite. In case the synthesis of a biomass precursor was blocked under these settings, the applied knockout strategy was considered to directly couple target compound production to precursor synthesis and thus to growth in general.

**Fig. 6:**
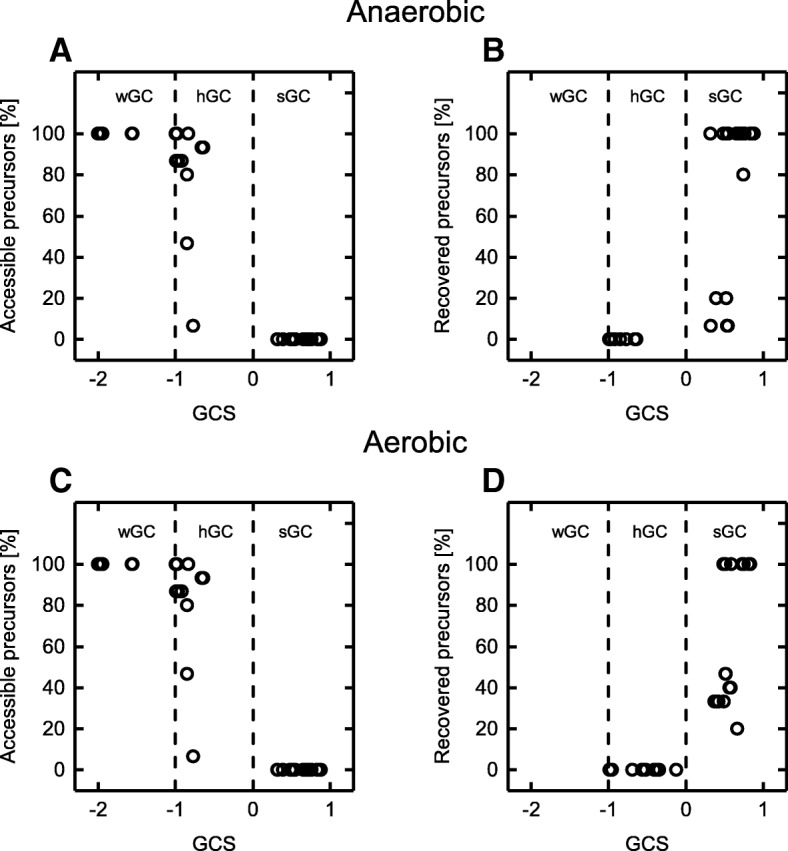
Biomass precursor availabilities for all identified GC strain designs under anaerobic and aerobic conditions. Standard ATPM requirements (**a**, **c**) and an unbounded, reversible ATP hydrolysis reaction (**b**, **d**) were employed. The vertical dashed lines separate the GCS range into three regions denoting wGC, hGC and sGC

Sixteen biomass precursors were derived from the left-hand-side of the biomass formation reaction included in the *E. coli* core reconstruction. In Fig. 6, the percentage of accessible precursors for each identified strain design leading to GC is plotted against the GCS, not distinguishing between the number or reaction deletions or metabolites coupled to growth. For all strain designs showing a GCS below − 1, thus being of type wGC, 100% of the biomass precursors were still accessible. This contradicts the principle of a direct coupling between biomass precursor and product synthesis but is actually trivial since for wGC strategies product synthesis is only enforced above a certain threshold growth rate (cf. Fig. 1). Likewise, this principle cannot explain product formation at zero growth for sGC. However, each identified sGC intervention strategy for anaerobic conditions resulted in blockage of *all* biomass precursors. Under aerobic conditions, this fraction was lower but still considerable. Only among the hGC strategies, a partial precursor blockage was found along with designs that had no effect on precursor availability at all. In none of the identified hGC solutions the synthesis of all biomass precursors was blocked.

Motivated by the observed increase in coverage and strength of GC strategies upon decreased ATP demand (Fig. 5), we wanted to further understand if and how the ATP metabolism might be a factor in establishing GC. To this end, we tested the biomass precursor availabilities for all identified strain designs allowing a reversible and completely unlimited flux through the ATPM reaction (Eq. 1). The consequence of this relaxation of the ATPM flux constraint is an unrestricted generation of ATP from ADP and free phosphate. While in all hGC cases (− 1 < GCS < 0), none of the precursors could be recovered, i.e., made accessible again by this relaxation, the synthesis of *every* blocked biomass precursor was restored for roughly 60 and 80% of the sGC strain designs (GCS > 0) under aerobic and anaerobic conditions, respectively (Fig. 6b and d).

### The effects of relaxing cofactor balances on growth-coupling strain designs

The investigation of biomass precursor availability in the GC mutants indicated that an enforced production of the target compound (sGC) is likely due to a global metabolic necessity rather than caused by a strict dependence of the synthesis of a particular biomass precursor on target compound production. Moreover, ATP scarcity seemed to be a metabolic trigger for GC in those sGC cases in which the synthesis of any biomass precursor was blocked by the intervention strategies. To challenge this hypothesis, the GCS of a GC strain design was investigated upon relaxing the directionality constraint of the ATPM equation (cf. Eq. 1) thereby enabling the model to freely phosphorylate ADP to ATP and vice versa. Since the ATP metabolism is interconnected with the redox cofactor and cross-membrane proton balance, e.g., via the electron transport chain and ATP synthase, a free NAD(P)H/NAD(P)^+^ generation and proton transport over the cell membrane were additionally tested for their effects on the GCS. To simulate this, the NADH and NADPH NGAM reactions (Eqs. 2–3) were reintroduced and a new proton translocation reaction was added:

4 $$ {H}_{ex}^{+}\leftrightarrow {H}_{in}^{+}. $$

Here, the indices *ex* and *in* locate the H^+^ protons to the extracellular and intracellular compartment, respectively. Both reactions were unbounded, i.e., allowed to carry any flux. All identified GC strategies and their GCS values under all investigated conditions are provided in the Additional files 1 and 2.

**Fig. 7:**
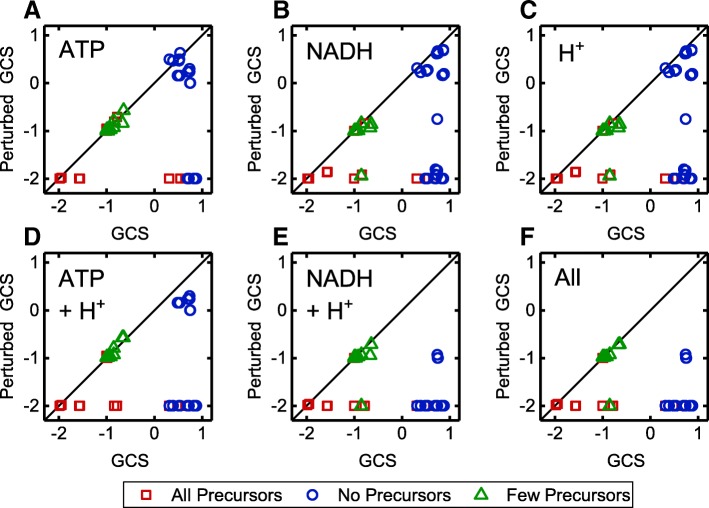
GCS of identified strain designs for anaerobic conditions and the corresponding GCS under certain relaxations. Relaxations concern ATPM (**a**), NADH/NAD conversion (**b**) and H^+^ translocation constraints (**c**) or combinations of those (**d**-**f**). The different colors or symbols relate to Fig. 6 showing the accessibility of biomass precursor for the same strain designs is shown. Red squares, blue circles and green triangles symbolize designs that allow for the synthesis of all, no or a reduced number of biomass precursors, respectively

For anaerobic conditions, GC was completely suppressed for all but two strategies by relaxing either the ATP balance, the NAD(P)H/NAD(P)^+^ conversion, the proton exchange or a combination of these strategies (Fig. 7). These two resistant strategies coupled formate to growth by forcing the carbon flux through the anchor reaction catalyzed by the pyruvate formate lyase, which splits pyruvate to formate and acetyl-CoA. However, the GCS of these strategies decreased when relaxing the constraints on cofactor generation and proton export.

**Fig. 8:**
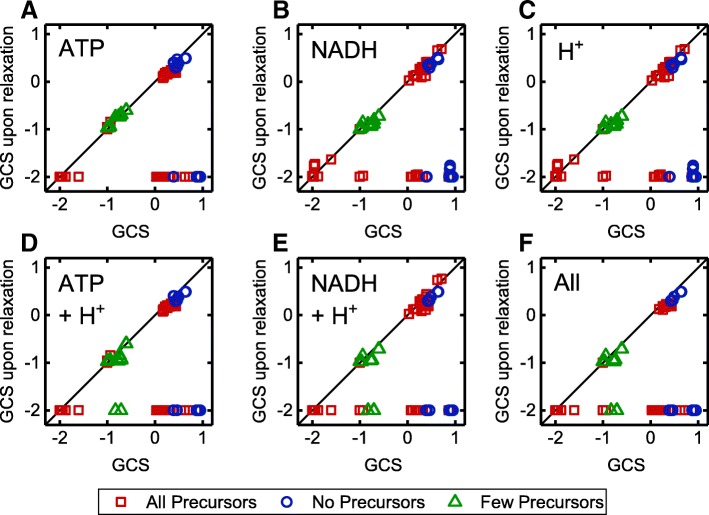
GCS of GC strain designs for aerobic conditions and the corresponding GCS under certain relaxations. Relaxations concern ATPM (**a**), NADH/NAD conversion (**b**) and H^+^ translocation constraints (**c**) or combinations of those (**d**-**f**). The different colors or symbols relate to Fig. 6 showing the accessibility of biomass precursor for the same strain designs is shown. Red squares, blue circles and green triangles symbolize designs that allow for the synthesis of all, no or a reduced number of biomass precursors, respectively

Disclosure of the basic coupling principles was impeded by the interrelatedness of redox cofactor, ATP and H^+^ balancing. For example, GC of lactate synthesis was abolished in most designs by relaxation of either ATP/ADP, NADH/NAD^+^ conversion or a free proton translocation. The basic coupling principle for this reduced metabolite is however NADH reoxidation, achieved by the reduction of pyruvate to lactate. Consequently, growth-coupling is abolished upon opening the NADH balance. Relaxation of the ATP and proton balance had the same effect as it fuels flux through the NADH transhydrogenase, which couples NADH oxidation and NADPH reduction to proton import. The formed NADPH is oxidized in biomass forming reactions making NADH re-oxidation by lactate dehydrogenase activity superfluous. Under standard conditions NADH transhydrogenase activity is limited by the cell’s potential to maintain a proton gradient over the cell membrane. In contrast, GC of ethanol was only abolished when free proton exchange was enabled. That was not expected as ethanol and lactate share almost the same synthesis pathway and as ethanol is more strongly reduced than lactate. Apparently, GC of ethanol was achieved in these designs by coupling the intracellular proton balance to the ethanol-proton symporter activity. As all intervention GC strategies for ethanol included the deletion of the ATP synthase, proton export via a reversed ATP synthase activity under relaxed ATP turnover conditions was not possible. GC of pyruvate was diminished by both free proton transport and ATP/ADP conversion. Inspection of the flux distribution under relaxed ATP/ADP conversion conditions revealed that excess ATP was used to drive the ATP synthase as proton exporter. Consequently, pyruvate secretion was enforced by the need to balance intracellular protons as was the case for ethanol. For aerobic conditions, relaxation of single or combinations of the tested constraints relieved GC for all wGC and most hGC strategies, as well. Again, formate was the only metabolite that was hard-coupled to growth by forcing flux through the pyruvate formate lyase anchor reaction. However, under aerobic conditions, this strategy is not of any relevance due to the pyruvate formate lyase’s sensitivity to oxygen [30]. Surprisingly, more than 50% the sGC strategies were not affected by alleviating cofactor and proton supply although in most of these cases all biomass precursors were accessible without enforced product synthesis (Fig. 8). Inspection of the robust strategies showed that coupling of metabolites of the upper central carbon metabolism was achieved by prohibiting phosphoenolpyruvate (PEP) conversion in the lower central carbon metabolism by deletion of PEP carboxykinase and pyruvate kinase, as well as the elimination of a cyclically operating pentose phosphate pathway, which would allow complete oxidation of the substrate to CO_2_. In vivo, this strategy might not be specific but could enforce the secretion of any upper central carbon metabolite. In our simulations, this was prohibited as only export reactions of lactate, ethanol, and formate were included in the model and the formation of these fermentation products was prevented by further reaction deletions in the GC strategies. In the remaining designs the metabolism was curtailed in a way that forced glucose oxidation through metabolic anchor reactions, here, transketolase, transaldolase or fructose bisphosphate aldolase, splitting the substrate into the target compound and an essential central carbon metabolism intermediate. As for the formate coupling strategies, the GCS of these strategies, although not completely abolished, was significantly reduced in most strategies upon relaxation of cofactor turnover and proton exchange. For the complete statistics corresponding to Figs. 7 and 8, we refer to Additional file 4: Tables S4 and S5.

### Growth-coupling affects the energy hierarchy of metabolites

The examination of the biomass precursor availability in the GC mutant strains and the influence of the ATP and NAD(P) H turnover and proton exchange on GC gave a first indication of the importance of the balancing of redox and energy cofactors. To further unravel the interdependency between GC and the cellular energy metabolism, the parameter ‘ATP synthesis capability’ (ATPsc) was defined. ATPsc assesses the contribution of the product synthesis to the global provision or consumption of ATP (cf. Methods section). The ATPsc represents the change in the maximal flux through the ATPM reaction when product synthesis is increased by 1 mmol g^− 1^ h^− 1^. It is furthermore practical to normalize ATPsc with the number of carbon atoms of the target product yielding the ATPsc per carbon (ATPcsc).

**Fig. 9:**
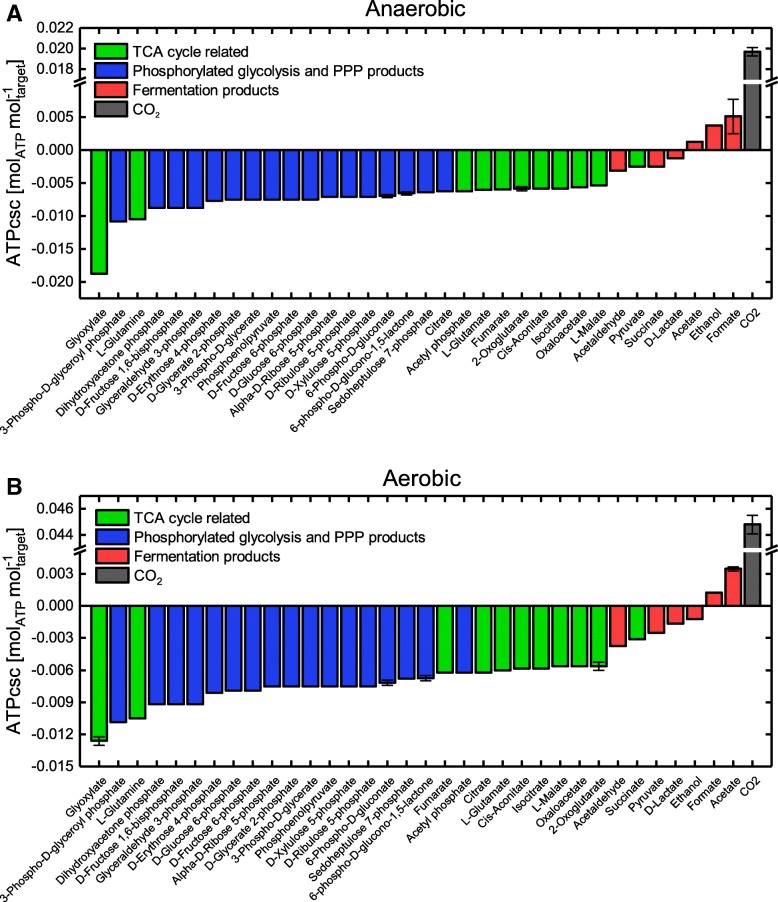
ATP synthesis capability values normalized by the number of carbon atoms (ATPcsc) for several metabolites of the central carbon metabolism. The *E. coli i*AF1260 core metabolic model was employed under anaerobic (**a**) and aerobic (**b**) conditions using glucose as the sole carbon and energy substrate. The order of the metabolites according to the ATPcsc value depicts the energy hierarchy of metabolites. Error bars denote the standard deviation of ATPcsc calculations at different growth rates spanning the feasible range of growth states. The color code links the metabolites to glycolysis and pentose phosphate pathway (PPP) (blue), TCA cycle (green) and fermentative pathways (red), respectively

For the *E. coli i*AF1260 core model the ATPcsc of CO_2_ is the highest followed by those of fermentation and overflow metabolites such as ethanol, lactate, succinate or acetate under anaerobic as well as aerobic conditions (Fig. 9). An almost identical energy hierarchy of metabolites is computed using the *E. coli i*JO1366 genome-scale model (Additional file 3: Figure S5), thus pointing to the fundamental nature of the ATPcsc value for the microbial metabolism. In line with the common understanding of the mechanisms of fermentation pathways [31, 32], this hierarchy correctly reflects the order of metabolites secreted by this organism under oxygen-limited or carbon excess conditions. Hence, we used the energy hierarchy as a measure for quantifying the ATP gain from product synthesis relative to other possible side products.

We hypothesized that growth coupling is induced or enhanced by the set of reaction deletions computed by gcOpt that deletes more efficient ATP generation routes so that the ATP yield of the target compound synthesis pathway excels those of residual ATP forming pathways. The observation that the ATP synthase was a frequent knockout target under aerobic conditions, diminishing ATP generation via oxidative phosphorylation, is in line with this hypothesis. Such a causality would become apparent by an increase of the ATPcsc in the mutant models and, in turn, an elevated rank of the target product in the energy hierarchy of metabolites. To test this assumption, the ATPcsc was calculated for a selection of 36 central metabolites of the wild-type *E. coli i*AF1260 core model and every identified GC intervention strategy using standard.

NGAM requirements. Energy hierarchies of metabolites for each GC strain design were arranged such that the metabolite with the highest ATPcsc value was ranked position one.

**Fig. 10:**
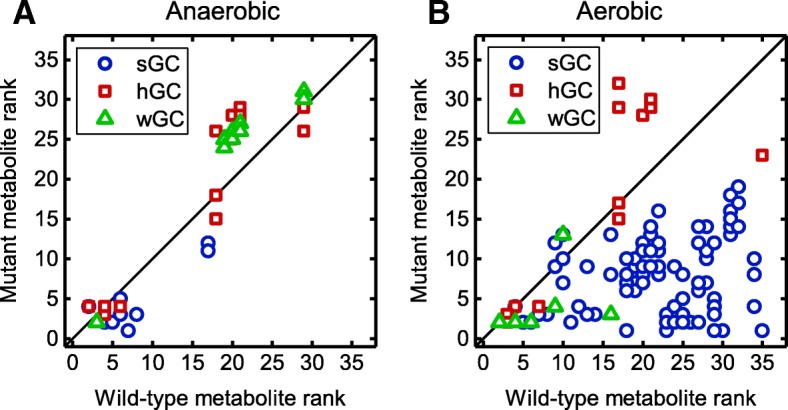
Rank of target metabolites in the energy hierarchy of energy for wild-type and GC mutant strains. Comparison of the rank of GC target products of the *E. coli i*AF1260 core model in the energy hierarchy of metabolites of GC mutants and the wild-type under anaerobic (**a**) and aerobic (**b**) conditions. The hierarchy is based on the ATPcsc values

Under anaerobic and aerobic conditions, for most sGC strategies the target compound’s rank in the hierarchy of metabolites increased compared with wild type conditions (Fig. 10). This was reversed for the wGC and hGC designs, in which the majority of target products faced a reduction of the energy hierarchy rank. For aerobic conditions (Fig. 10b), the upward shift of the target compounds in the energy hierarchy was considerably more pronounced than under anaerobic conditions (Fig. 10a) and for 30% of the sGC strain designs the target compound was even ranked second or first, thus, in case of the latter, surpassing CO_2_ as the most beneficial production pathway for generating an excess supply of ATP.

## Discussion

Two major GC principles have been described in the literature, in which target production is enforced by either coupling it to the synthesis of biomass precursors or the balancing of energy and redox cofactors. Besides, the observation that yield and specific productivity is increased upon inducing an ATP futile cycle has been discussed as another principle [29, 33]. However, this latter interrelationship cannot be transferred to GC according to the findings shown in this work. Under anaerobic conditions, only a simulated *decrease* of the ATP consumption for cell maintenance processes led to an increased number of growth-coupled metabolites as the surplus of available ATP allowed for the secretion of compounds with higher energy contents such as phosphorylated intermediates. Identified GC strain designs also showed an enhanced GCS at reduced maintenance requirements. The prevalent ATP scarcity under anaerobic conditions may also be the reason for the observed stagnation of the GCS with increasing size of the intervention sets. A metabolic boundary, most likely ATP limitation, might confine the maximal ratio between the energetically disadvantageous product synthesis and growth. ATP supply is less critical in an aerobic setup due to a fully functional respiratory chain. This is reflected in the indifference of the GCS to alterations of energy and equally redox balances. Apparently, the better supply of redox and energy cofactors under aerobic conditions implies a superior metabolic capacity for product-growth coupling as was previously hypothesized [21]. Accordingly, we can phrase the following GC requirement:

*Generation of strong coupling of growth and product synthesis is ultimately limited by the cell’s natural capacity to generate energy equivalents in form of ATP.*

This may be seen contradictory to the findings shown in Fig. 5 where it can be observed that for a high as well as low ATP maintenance demands GC strain designs with similar mean GCS values can be obtained under *aerobic conditions*. In fact, this simply reflects the ability of the cell under aerobic conditions to compensate for the energy-demanding product secretion by flexible adjusting the metabolic pathway usage to generate ATP via substrate level phosphorylation sustaining its ATP demand, at least in the range tested here.

This became also apparent from the ATPcsc values of GC mutants. For aerobic conditions, most target products soared in the energy hierarchy ranking. Thus, their synthesis pathway became a more, or in some case *the* most advantageous metabolic route to regenerate ATP in terms of an optimal compromise between carbon usage and ATP yield.

In summary, the here presented, rigorous calculations of GC strategies using gcOpt confirmed previously published results [21, 25]:

-
**Principle 1:** *Feasibility of GC holds for a wide range of metabolites.*

Yet, GC of energy-rich as well as oxidized metabolites and the ability to reach high coupling strengths was found to be limited to aerobic conditions. The apparent, global feasibility of GC pronounces the applicability of this concept for *any* microbial strain engineering project aiming to increase productivity and yield. This observation of global feasibility is primarily based on reaction deletion strategies. Due to the existence of isozymes, promiscuous enzymes and multiprotein complexes, the number of necessary gene deletions may significantly differ from the number of suggested reaction deletions. The realization and implementation of GC strain designs in vivo may thus be hampered. In turn, the application of a strain design framework based on a heuristic genetic optimization algorithm proved to overcome this limitation [34]. It allows for the identification of *gene* deletion strategies leading to GC by employing the gcOpt principle and specifically considering the logical links between genes and reactions given by the model-inherent gene-protein-reaction relations.

However, the intuitive approach towards the generation of GC is to enforce an obligatory dependence of the synthesis of one or more essential biomass precursors on target compound production. However, such a GC criterion can only explain or lead to hGC characteristics, in which product synthesis is strictly bound to biomass formation. In fact, we found that the majority of hGC strategies blocked the synthesis of one or more biomass precursors at zero product formation. The concept can be broadened to the following principle and was indeed evident for 50% of all aerobic hGC and sGC strategies.

-
**Principle 2:** *Linking product synthesis to reactions essential for any steady state flux distribution on the chosen carbon source results in holistic and strong GC. Those reactions include but are not restricted to biomass precursor forming reactions.*

However, our analysis also highlights the balancing of global cofactor as an additional, important criterion for establishing or enhancing GC, hence leads us to a specialized version of the third principle.

-
**Principle 3:** *Reconfiguration of the metabolic network rendering the product synthesis pathway the superior ATP supply route is one major principle for generating strong growth coupling or enhancing the GCS.*

Here again, this principle seems to contradict the findings in Fig. 5 stating that the ATP demand does not affect the *identification* of strain designs with increasing GCS values. Principle 3, however, refers to the majority of (s)GC strategies *identified* under standard conditions for which lowering the ATPM demand a posteriori weakens the GC strength of the respective mutants.

In any way, principle 3 can be intuitively inferred from the observation that the ATP synthase is a frequent knockout target in aerobic GC strategies and more quantitatively be described with the rise of the target metabolite in the energy hierarchy. This is supported by the observation of Jensen & Michelsen [35] that an ATP synthase deficient *E. coli* strain shifts the flux distribution towards substrate-level phosphorylation pathways, i.e., glycolysis and TCA cycle, and the secretion of correlated metabolites. We conclude that, for *E. coli* strains, ATP synthase deletion forms a basis for GC under aerobic conditions whereas additional knockouts enforce specificity of product secretion as can be derived from the steadily increasing mean GCS with increasing genetic interventions. Exceptions from this pattern are fermentation products or products exported via proton symporters, for which GC is induced by disrupting alternative NADH re-oxidizing or proton translocating pathways.

## Conclusion

The formulation of a mixed integer linear program which maximizes the minimally guaranteed production rate of a target metabolite at a medium, fixed growth rate, realized in gcOpt, has been shown to yield attractive strain designs with growth-coupled target production. One advantage of the identified growth-coupling (GC) strategies, at least of the investigated test cases, is the existence of only one feasible target production rate at maximal growth rather than a range of possible production rates.

Generally, metabolic network reconfigurations that render product secretion into a carbon drain necessary for metabolic activity might be the more robust GC approach as it is independent of cofactor and proton balancing that might vary under different growth conditions. However, such a coupling might not be possible for all metabolites. Our analysis revealed that coupling product formation to cofactor supply or turnover not only enhances the GCS of the former strategies but also seems to be globally applicable to any metabolite. In contrast to the more comprehendible and manually applied concept of coupling target metabolite production to biomass precursor synthesis, metabolic designs that are based on such cofactor balancing are hard to derive manually. This is mainly due to the complex interconnectedness of energy and redox cofactors within metabolic networks. Accordingly, we argue that computer-aided network analysis can accelerate the development of strain designs strictly coupling production to microbial growth by predicting effective GC strategies with a reasonable number of gene deletions.

## Methods

### Formulation of gcOpt

gcOpt is geared to existing multi-level optimization frameworks and their lower-level formulations optimizing an engineering objective by searching for appropriate sets of reaction deletions [15, 16]. gcOpt maximizes the minimally guaranteed production rate *v*_*t*_ of a target compound *t* for a fixed growth rate μ_fix_. The corresponding bi-level optimization problem is formulated as follows:

where $\overline{y}$ is a boolean vector indicating for each reaction *i* ∈ *R* within the reversible metabolic model if *i* is inactive (0) or active (1). With constraint (6), the size of the reaction deletion set is limited to *K* interventions. Note that the gcOpt formulation requires the splitting of all reversible reaction of the original model into irreversible forward and backward reactions. This results in an irreversible metabolic model containing *N* reactions with strictly positive fluxes (Eq. 10). The flux value of each irreversible reaction is contained in the vector $\overline{v}$. Steady state mass balances of intracellular metabolites are assured by Eq. 8. Here, *S* ∈ *ℝ*^|*M*| × |*N*|^ is the stoichiometric matrix where each non-zero value *S*_*j*, *i*_ denotes the stoichiometric coefficient of metabolite *j* ∈ *M* participating in reaction *i* ∈ *N*. Each flux v_i_ is constrained by lower and upper bounds ${\mathrm{v}}_{\mathrm{i}}^{\mathrm{lb}}$ and ${\mathrm{v}}_{\mathrm{i}}^{\mathrm{ub}}$, respectively, or set to zero if y_k_ indicates a knockout (Eq. 9). The connection between a reversible reaction *k* and one of its irreversible counterparts *i* is kept in the mapping matrix *B* ∈ {0, 1}^|*N*| × |*R*|^ by *B*_*i*, *k*_ = 1. Since the biomass reaction v_bm_ is fixed to μ_fix_ by Eq. 11, μ_fix_ needs to be lower than the maximally achievable biomass formation rate ${\mathrm{v}}_{\mathrm{bm}}^{\mathrm{max}}$.

Solving the nested mixed-integer optimization problem (Eq. 5-11) using linear programming solvers is intractable [15]. By virtue of the linearity of the outer and inner objective function as well as the posed equality and inequality constraints, the bi-level optimization problem (Eq. 5-11) can be recast to a single-level MILP by exploiting the strong duality theorem in linear programming [36]. For gcOpt, the reformulation was done as described by Tepper & Shlomi [16] but adapted to the differently formulated objective functions.

In this work, core models of the central carbon metabolism introduced by Trinh et al. [4] and Orth et al. [37] as well as the advanced metabolic model *i*JO1366 of *E. coli* K-12 MG1655 [38] were used. To improve the tractability of strain designs computations, the solution space of the genome-scale model *i*JO1366 (1366 genes, 2251 reactions and 1136 metabolites) was reduced following a preprocessing routine similar to a protocol of Feist et al. (2010), which was integrated into the gcOpt framework. More specifically, essential as well as exchange, diffusion, transport and spontaneous reactions were excluded from the set of possible target reactions for deletion. Furthermore, reactions contained in the subsystems cell envelope biosynthesis, membrane lipid metabolism, murein biosynthesis, tRNA charging and glycerophospholipid metabolism were also not regarded as deletion targets. In addition to reducing the solution space of the problem posed by gcOpt, the actual number of reactions within the considered metabolic model was trimmed by entirely removing all reactions unable to carry any flux. These so called blocked reactions were identified by flux variability analysis [39] by means of a maximum and minimum flux equal to zero.

The gcOpt framework was implemented in MATLAB 2016b and is freely available on GitHub (https://github.com/Spherotob/gcOpt). For solving the single-level MILP derived from problem (1), the Gurobi Optimizer (7.0.2, Gurobi Optimization, Inc.) was utilized. All computations in this work were conducted on a Windows 7 machine with a maximum configuration of 16 GB of RAM and an AMD FX-8350 Eight-Core (à 4.00 GHz) processor.

### Quantification of the growth-coupling strength

To quantify and compare the GC level or strength of microbial strain designs we sought for a distinct measure. We required this measure to simultaneously (1) reflect the actual coupling strength in terms of the position of the lower production rate bound, (2) the yield at physiologically relevant growth conditions, as well as (3) the qualitative coupling type. Hence, we defined GCS, a novel measure for the growth coupling strength based on the production envelope representation. As visualized in Fig. 4, the ratio between the area *IA* below the lower production rate bound and the total area *TA* under the upper production rate hull curve in the production envelope of a strain design is the core of the GCS and fulfills requirement (1). This expresses the principle that the flux modes with the lowest yields are sequentially made inaccessible the stronger the coupling between growth and product synthesis becomes. To account for point (2), the minimally guaranteed target product yield at maximal growth ${Y}_{min}^{\mu_{max}}$ divided by the theoretical maximal yield *Y*_*max*_ is considered as a factor in the formula for the GCS (Eq. 6.1-6.3). For strain designs with similar GC levels according to an evaluation of the production envelope areas, this factor promotes those that predicts high yields at physiologically relevant growth rates. To be able to directly distinguish the GC types sGC, hGC and wGC as stated in requirement (3), the intersection of the lower production rate boundary and the growth axis was further integrated. Following this, the GCS is finally calculated as follows:

Here, ${v}_{p,\mathit{\min}}^{\mu =0}$ and ${\mu}_{max}^{Y=0}$ are the minimal target production rate at zero growth and the maximal growth rate at zero production, respectively. GCS increases with increasing GCS values and the three GC types are defined by distinct GCS ranges. To allow for an immediate distinction of the GC types, GCS of hGC and wGC strategies are normed by considering one and two as an additional subtrahend in Eq. 12. Thus, a GCS between −2 and − 1 denotes wGC, the interval [−1, 0] indicates hGC and GCS > 0 implies sGC. Hence, the GCS parameter enables both a qualitative classification and a quantitative ranking of GC strain designs. In the data evaluation process, strategies with GCS ≤ − 1.975 and between −0.975 and -1 were considered to confer no coupling.

### Probing the biomass precursor availability

To evaluate the capability of a metabolic network to synthesize a particular biomass precursor the biomass synthesis equation was singularized into separate, independent reactions, i.e., for each single reactant M with stoichiometric coefficient υ_m_ in the biomass equation a new, unbounded reaction of the form υ_m_M → was defined. Similarly, for each product N and stoichiometric coefficient υ_n_ a reaction → υ_n_N was added to the model. Likewise, if the consumption of a precursor was coupled to the production of a certain compound, e.g. ATP and ADP, both were linked in a mass-balanced reaction of the form υ_m_M → υ_n_N. The original biomass equation was erased from the model. The availability of a biomass precursor of this original equation was then tested by setting up a linear program that maximized the related singularized reaction subject to all mass balance, substrate uptake and thermodynamic constraints of the original model. A maximal achievable flux of zero, implies loss of the metabolic capacity to synthesize the respective precursor and hence, this precursor is inaccessible. For cases with positive maximal fluxes, the synthesis of the precursor is not impaired.

### ATP synthesis capability

The ATP synthesis capability (ATPsc) parameter was created to deduce the influence of byproduct secretion on the synthesis and provision of the cellular energy equivalent. For a given metabolic network and production rate of a target compound, the ATPsc describes the change of the maximal flux through the reaction ATPM in response to a change of the production rate of the target chemical. Mathematically, the ATPsc is defined by the derivative dv_ATPM max_/dv_target exchange_ with v_ATPM max_ being the maximal flux through the ATPM reaction (Eq. 1) computed by linear programming and a metabolic network constrained with a target product exchange rate fixed to values between zero and the maximal flux. Graphically, ATPsc can be determined by plotting the ATPM flux values against the product exchange rate and calculating the slope of the graph (Additional file 3: Figure S4). Using the *E. coli i*JO1366 metabolic model, the ATPsc was calculated for a range of metabolites of the central carbon metabolism for low production rates. Thus, the resulting ATPsc values correspond to the differences in maximal accessible excess ATP between inactive and active metabolite secretion. For each metabolite, the ATPsc was calculated for a range of accessible growth rates. Additional file 3: Figure S4 shows the results for anaerobic and aerobic conditions. Here, mean ATPsc values were normalized by the number of carbon atoms of the target compound (termed ATPcsc).

## Additional files

Additional file 1: GCS values for the identified GC strategies of all metabolites of the central carbon metabolism under anaerobic conditions. GC strategies with different numbers of maximally allowable reaction deletions were computed using gcOpt with the *E. coli i*AF1260 core model. GCS values are additionally provided for an unconstrained ATP/ADP and NADH/NAD conversion, an unhindered proton translocation reaction, as well as combinations of these relaxations. The dataset includes the respective reaction and gene deletions for each identified strategy. (XLS 87 kb)

Additional file 2: GCS values for the identified GC strategies of all metabolites of the central carbon metabolism under aerobic conditions. GC strategies with different numbers of maximally allowable reaction deletions were computed using gcOpt with the *E. coli i*AF1260 core model. GCS values are additionally provided for an unconstrained ATP/ADP and NADH/NAD conversion, an unhindered proton translocation reaction, as well as combinations of these relaxations. The dataset includes the respective reaction and gene deletions for each identified strategy. (XLS 107 kb)

Additional file 3: **Figure S1.** Succinate production envelopes of strain designs identified by gcOpt (A) and OptKnock (B) under aerobic conditions. **Figure S2.** Lactate production envelopes of strain designs identified by gcOpt (A) and OptKnock (B) under anaerobic conditions. **Figure S3.** Ethanol yield spaces of GC strain designs identified by gcOpt in comparison to designs taken from literature. **Figure S4.** Relation between maximal ATP maintenance flux (ATPM) and the production rate of several metabolites under anaerobe and aerobe conditions. **Figure S5.** ATP synthesis capability values normalized by the number of carbon atoms (ATPcsc) for several metabolites of the central carbon metabolism. (DOCX 1805 kb)

Additional file 4: **Table S1.** Reaction deletions of GC strain designs identified by gcOpt in comparison to designs taken from literature. **Table S2.** Reaction deletions of GC strain designs identified by gcOpt and OptKnock for the production of succinate under aerobic conditions. **Table S3.** Reaction deletions of GC strain designs identified by gcOpt and OptKnock for the production of lactate under anaerobic conditions. **Table S4.** Effect of the relaxation of ATP/ADP and NADH/NAD^+^ conversion as well as the proton translocation on the GCS of the identified GC strain designs for anaerobic conditions. **Table S5.** Effect of the relaxation of ATP/ADP and NADH/NAD^+^ conversion as well as the proton translocation on the GCS of the identified GC strain designs for aerobic conditions. (DOCX 59 kb)

## Data Availability

The Matlab-based implementation of gcOpt is available on Github via https://github.com/Spherotob/gcOpt (DOI:10.5281/zenodo.1161712).

## Abbreviations

ADP: Adenosine diphosphate
ATP: Adenosine triphosphate
ATPcsc: Carbon normalized ATP synthesis capability
ATPM: ATP maintenance
ATPsc: ATP synthesis capability
GC: Growth-coupling
GCS: Growth-coupling strength
hGC: Holistic growth-coupling
MCS: Constrained minimal cut-set
MCS: Minimal cut-set
MMF: Minimal metabolic functionality
NAD^+^: Oxidized nicotinamide adenine dinucleotide
NADH: Reduced nicotinamide adenine dinucleotide
NGAM: Non-growth associated maintenance
PEP: Phosphoenolpyruvate
sGC: Strong growth-coupling
wGC: Weak growth-coupling
