# Supplementary material for: Determination of growth-coupling strategies and their underlying principles
Source: BMC Bioinformatics. 2019 Aug 28;20:447. doi: 10.1186/s12859-019-2946-7 (PMC6714386; doi:10.1186/s12859-019-2946-7)
Supplement: Supplementary file 3 — Figure S1. Succinate production envelopes of strain designs identified by gcOpt (A) and OptKnock (B) under aerobic conditions. Figure S2. Lactate production envelopes of strain designs identified by gcOpt (A) and OptKnock (B) under anaerobic conditions. Figure S3. Ethanol yield spaces of GC strain designs identified by gcOpt in comparison to designs taken from literature. Figure S4. Relation between maximal ATP maintenance flux (ATPM) and the production rate of several metabolites under anaerobe and aerobe conditions. Figure S5. ATP synthesis capability values normalized by the number of carbon atoms (ATPcsc) for several metabolites of the central carbon metabolism. (DOCX 1805 kb) [file 12859_2019_2946_MOESM3_ESM.docx]

**Figures, Supplementary**

**
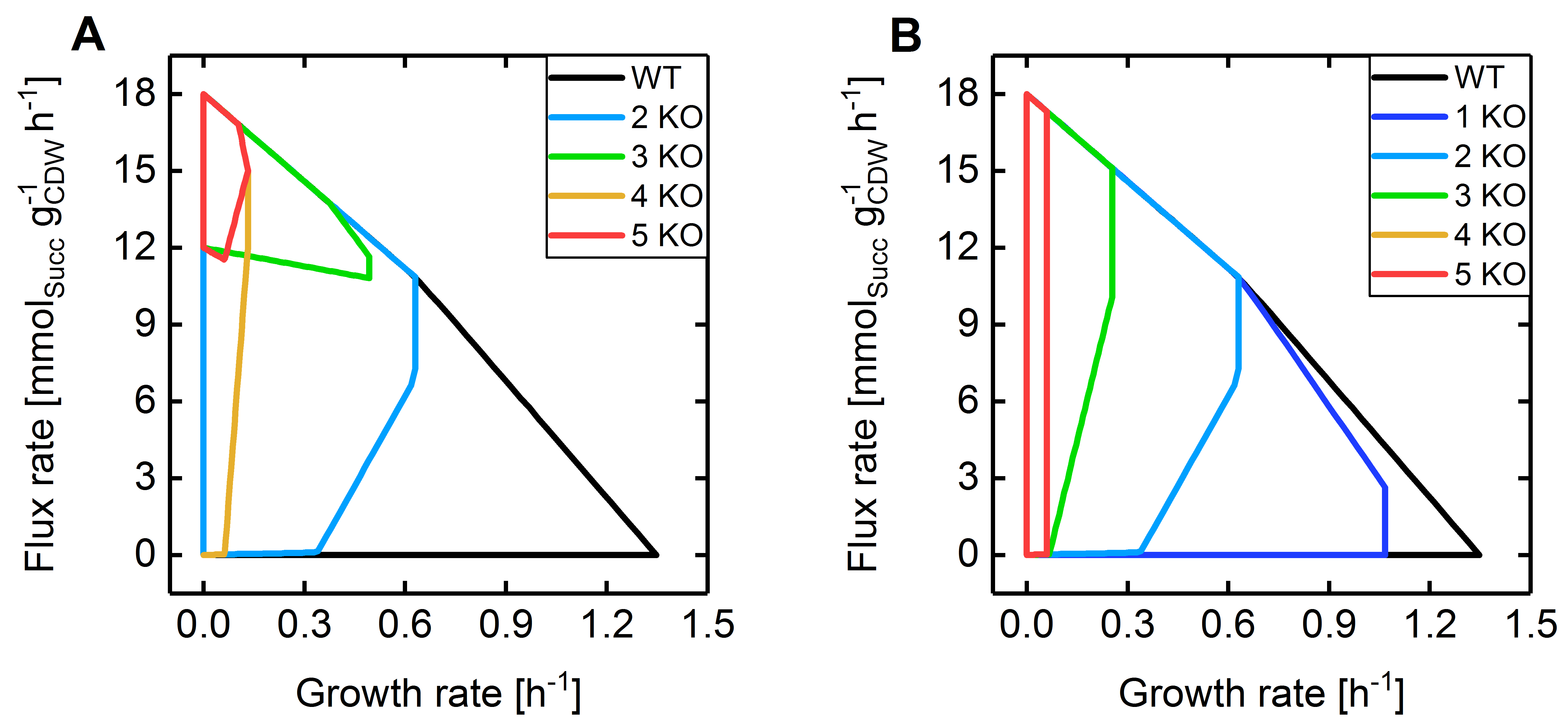
**

**Figure S1. Succinate production envelopes of strain designs identified by gcOpt (A) and OptKnock (B) under aerobic conditions.** Maximal intervention sizes between one and five reaction deletions were used to calculate GC strain designs employing the *E. coli* core model built by Trinh *et al.*[1]*.* Black lines denote the production envelopes of the wild-type. The maximal glucose uptake rate was constrained to 12 mmol g^-1^ h^-1^ for all respective simulations. A fixed growth rate $\mu_{fix}$ of 0.1 h^-1^ was applied for the gcOpt simulations. No GC strategy could be identified by gcOpt for a maximal intervention size of one. The quadruple (4 KO) and quintuple (5 KO) deletion strategy found by OptKnock overlap each other.

**
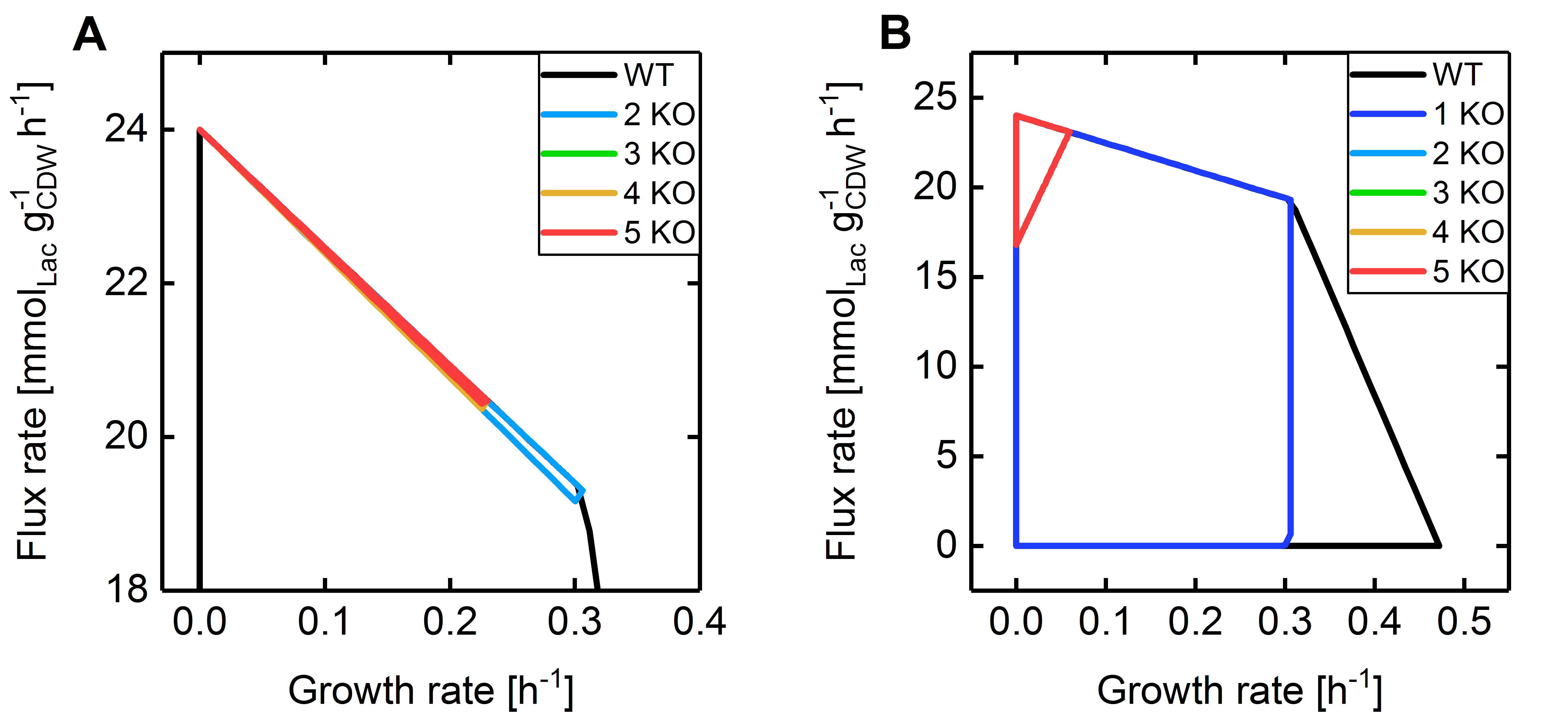
**

**Figure S2. Lactate production envelopes of strain designs identified by gcOpt (A) and OptKnock (B) under anaerobic conditions.** Maximal intervention sizes between one and five reaction deletions were used to calculate GC strain designs employing the *E. coli* core model built by Trinh *et al.*[1]. Black lines denote the production envelopes of the wild-type. The maximal glucose uptake rate was constrained to 12 mmol g^-1^ h^-1^ for all respective simulations. A fixed growth rate $\mu_{fix}$ of 0.1 h^-1^ was applied for the gcOpt simulations. No GC strategy could be identified by gcOpt for a maximal intervention size of one. GC strain designs from three to five reaction deletions found by gcOpt overlap each other. This is also the case for the OptKnock strategies from two to five reaction deletions.

| **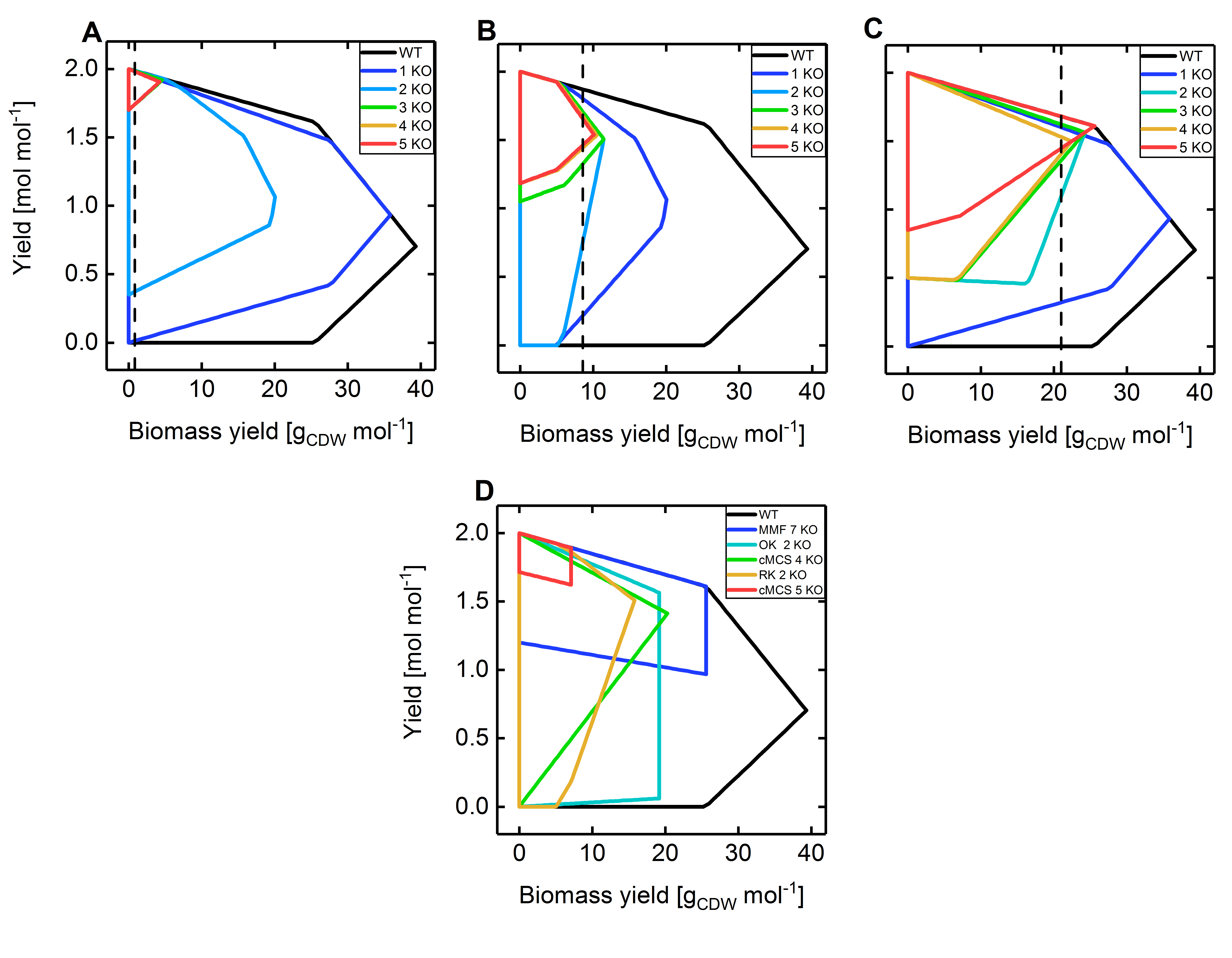** |
| --- |
| **Figure S3. Ethanol yield spaces of GC strain designs identified by gcOpt in comparison to designs taken from literature.** Maximal intervention sizes between one and five reaction deletions were used (A-C) and compared to several methods reported in the literature (D) [1, 2]. Black lines denote the production envelopes of the wild-type. The vertical black dashed lines mark the chosen fixed growth rates μ_fix_ for the respective computations (0.01 h^-1^ (A), 0.1 h^-1^ (B) and 0.25 h^-1^ (C)). The maximal glucose uptake rate was constrained to 12 mmol g^-1^ h^-1^ for all respective simulations. |

| **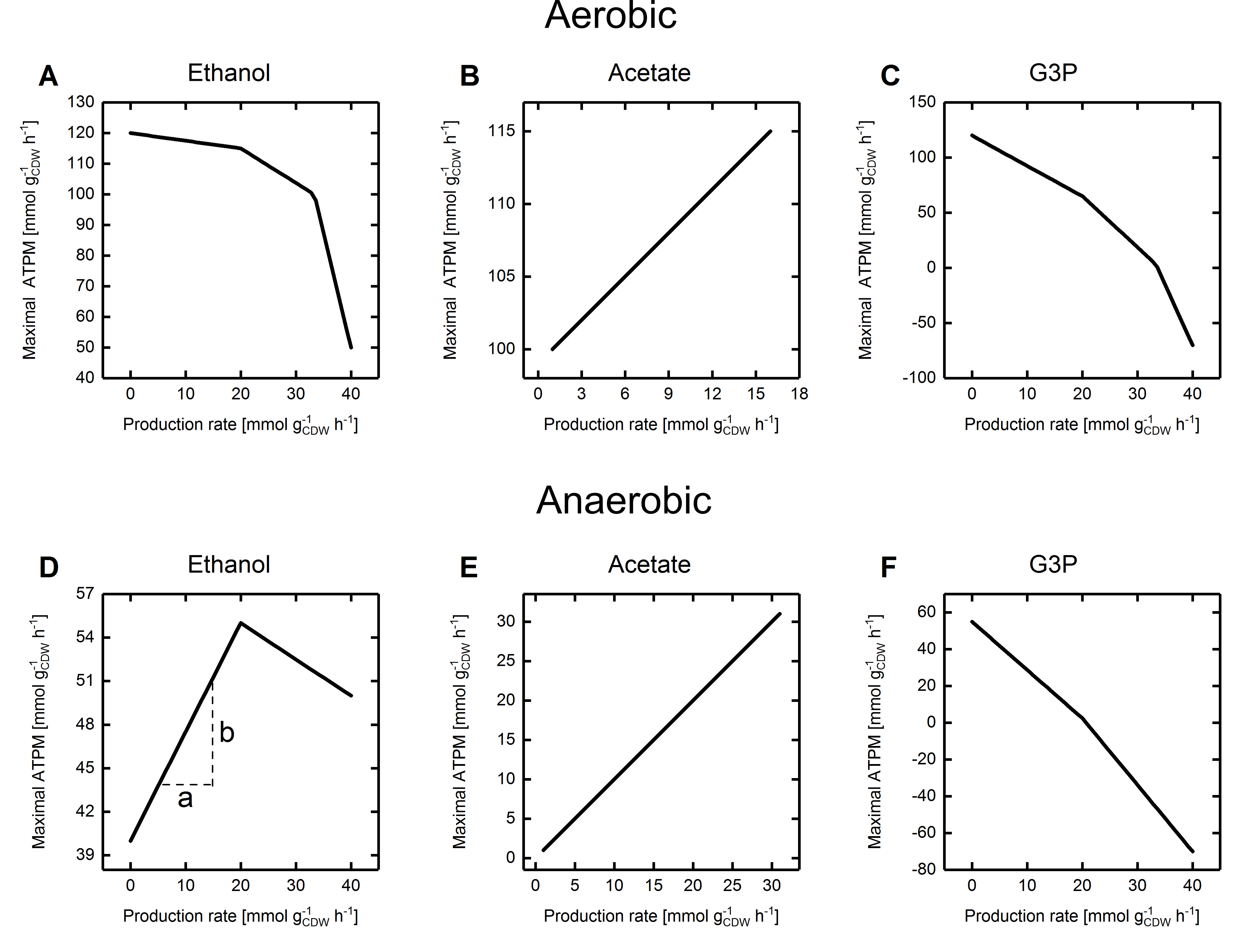** |
| --- |
| **Figure S4. Relation between maximal ATP maintenance flux (ATPM) and the production rate of several metabolites under anaerobe and aerobe conditions.** Maximally achievable ATPM was exemplarily computed for a range of production rate values for ethanol (A, D), acetate (B, E) and glyceraldehyde-3-phosphate (G3P) (C, F) under aerobic and anaerobic conditions using the *E. coli* *i*AF1260 core model and glucose as the sole carbon substrate. In (D), calculation of the gradient s is shown exemplarily by s = b/a, which is the basis for computing the ATP synthesis capability (ATPsc) as explained in chapter 4.4 in the main text. |

| **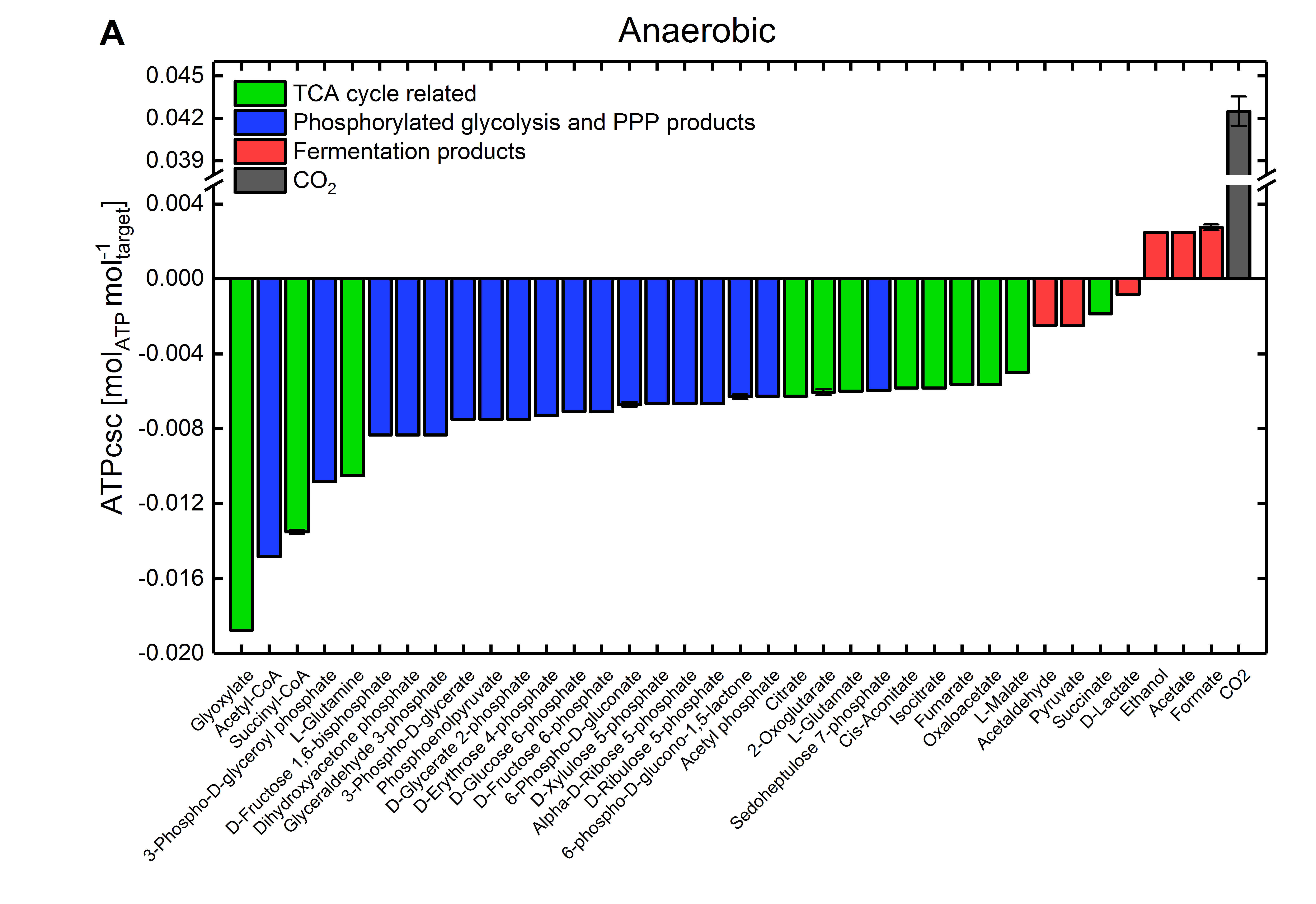** |
| --- |
| **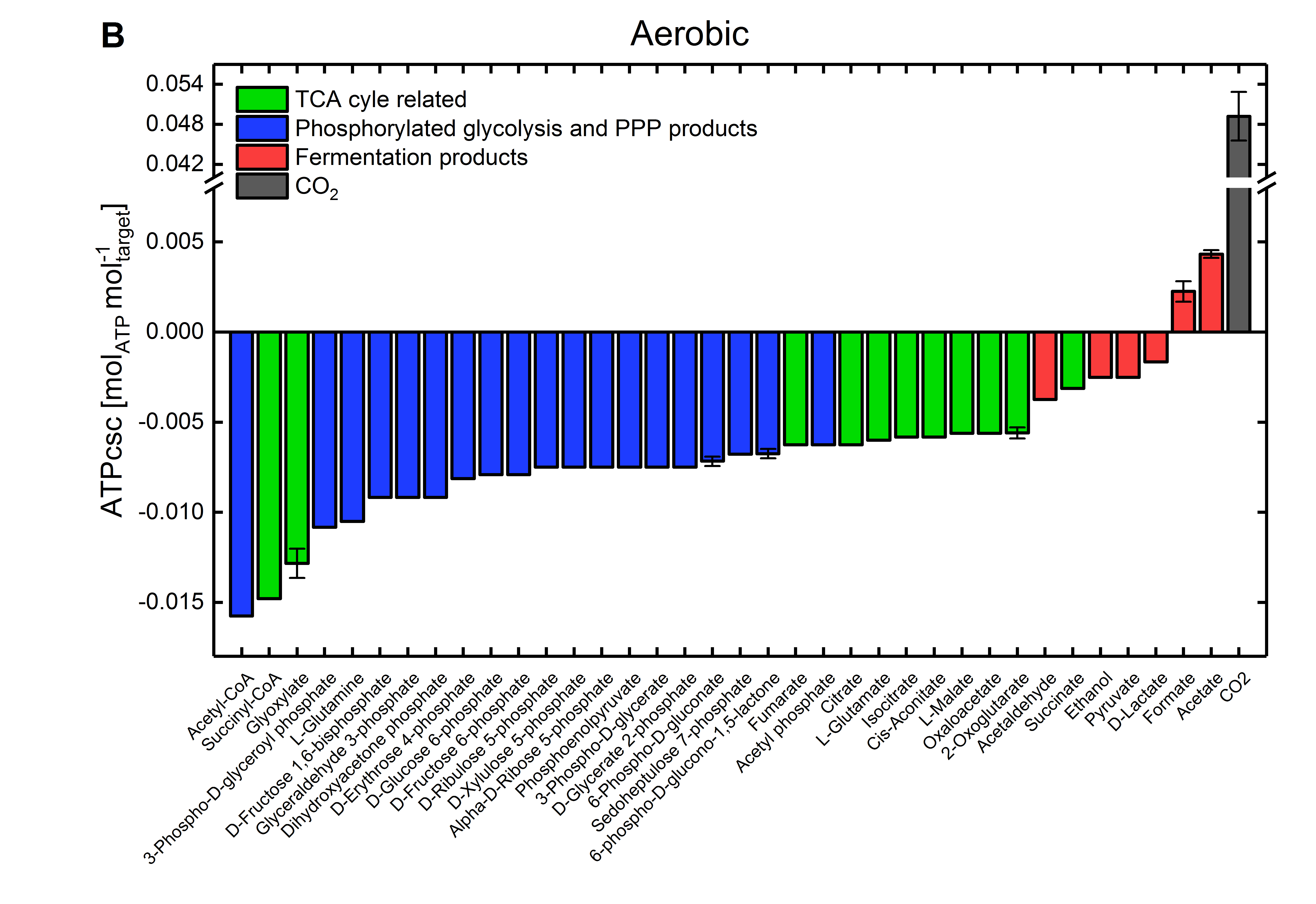** |
| **Figure S5. ATP synthesis capability values normalized by the number of carbon atoms (ATPcsc) for several metabolites of the central carbon metabolism.** The *E. coli i*JO1366 metabolic model was employed under anaerobic (A) and aerobic (B) conditions using glucose as the sole carbon and energy substrate. The order of the metabolites according to the ATPcsc value depicts the energy hierarchy of metabolites (cf. section 2.5 in the main text). Error bars denote the standard deviation of ATPcsc calculations at different growth rates spanning the feasible range of growth states. The color code links the metabolites to glycolysis and pentose phosphate pathway (PPP) (blue), TCA cycle (green) and fermentative pathways (red), respectively. |

**REFERENCES**

1. Trinh CT, Unrean P, Srienc F. Minimal *Escherichia coli* cell for the most efficient production of ethanol from hexoses and pentoses. Appl Environ Microbiol. 2008;74:3634–43. doi:10.1128/AEM.02708-07.

2. Hädicke O, Klamt S. Computing complex metabolic intervention strategies using constrained minimal cut sets. Metab Eng. 2011;13:204–13.
