# Supplementary material for: Determination of growth-coupling strategies and their underlying principles
Source: BMC Bioinformatics. 2019 Aug 28;20:447. doi: 10.1186/s12859-019-2946-7 (PMC6714386; doi:10.1186/s12859-019-2946-7)
Supplement: Supplementary file 4 — Table S1. Reaction deletions of GC strain designs identified by gcOpt in comparison to designs taken from literature. Table S2. Reaction deletions of GC strain designs identified by gcOpt and OptKnock for the production of succinate under aerobic conditions. Table S3. Reaction deletions of GC strain designs identified by gcOpt and OptKnock for the production of lactate under anaerobic conditions. Table S4. Effect of the relaxation of ATP/ADP and NADH/NAD+ conversion as well as the proton translocation on the GCS of the identified GC strain designs for anaerobic conditions. Table S5. Effect of the relaxation of ATP/ADP and NADH/NAD+ conversion as well as the proton translocation on the GCS of the identified GC strain designs for aerobic conditions. (DOCX 59 kb) [file 12859_2019_2946_MOESM4_ESM.docx]

**Tables, Supplementary**

**Table S1. Reaction deletions of GC strain designs identified by gcOpt in comparison to designs taken from literature.** Maximal intervention sizes between one and five reaction deletions (1-5 KO) were used to calculate GC strain designs using fixed growth rates μ_fix_ of 0.01 h^-1^, 0.01 h^-1^ and 0.25 h^-1^. The reaction deletions of each identified GC strategy are given according to the abbreviations from Trinh et al. [1].

| μ_fix_ = 0.01 h^-1^ | | | | |  | μ_fix_ = 0.1 h^-1^ | | | | |
| --- | --- | --- | --- | --- | --- | --- | --- | --- | --- | --- |
| 1 KO | 2 KO | 3 KO | 4 KO | 5 KO |  | 1 KO | 2 KO | 3 KO | 4 KO | 5 KO |
| ANA1 | GG2r | GG2r | GG6r | GG5r |  | GG2r | GG5r | GG3 | GG6r | GG2r |
|  | FEM3 | FEM1 | PPP8r | PPP8r |  |  | FEM7 | FEM3 | GLB2 | PPP3 |
|  |  | FEM3 | FEM1 | FEM1 |  |  |  | FEM7 | FEM3 | GLB1 |
|  |  |  | FEM3 | FEM3 |  |  |  |  | FEM7 | FEM3 |
|  |  |  |  | FEM5 |  |  |  |  |  | FEM7 |
|  |  |  |  |  |  |  |  |  |  |  |
| μ_fix_ = 0.25 h^-1^ | | | | |  | Literature | | | | |
| 1 KO | 2 KO | 3 KO | 4 KO | 5 KO |  | MMF [1]  (7 KO) | cMCS [2] (4 KO) | cMCS [2] (5 KO) | RB [2]  (2 KO) | OK [2] (2KO) |
| ANA1 | FEM1 | FEM1 | PPP8r | TCA6r |  | PPP1 | EDP1 | TRA5 | FEM7 | FEM5 |
|  | FEM3 | FEM3 | FEM1 | GLB1 |  | FEM2 | PPP8r | GG11 | GG2r | FC1r |
|  |  | FEM7 | FEM3 | FEM2 |  | FEM3 | FC1r | FEM3 |  |  |
|  |  |  | FEM7 | FEM3 |  | FEM7 | TRA5 | FEM7 |  |  |
|  |  |  |  | FEM7 |  | TCA10 |  | OPM4r |  |  |
|  |  |  |  |  |  | OPM4r |  |  |  |  |
|  |  |  |  |  |  | ANA2 |  |  |  |  |

**Table S2. Reaction deletions of GC strain designs identified by gcOpt and OptKnock for the production of succinate under aerobic conditions.** Maximal intervention sizes between one and five reaction deletions (1-5 KO) were used to calculate GC strain designs employing the E. coli core model built by Trinh et al. [1]. A fixed growth rate $\mu_{fix}$ of 0.1 h^-1^ was applied for the gcOpt simulations. The reaction deletions of each identified GC strategy are given according to the abbreviations from Trinh et al. [1].

| gcOpt | | | | |  | OptKnock | | | | |
| --- | --- | --- | --- | --- | --- | --- | --- | --- | --- | --- |
| 1 KO | 2 KO | 3 KO | 4 KO | 5 KO |  | 1 KO | 2 KO | 3 KO | 4 KO | 5 KO |
| - | TRA6 | TRA7 | TRA7 | TRA7 |  | TRA7 | TRA7 | TRA7 | TRA7 | TRA7 |
|  | TRA7 | EDP2 | TRA2 | TRA4 |  |  | FEM1 | TRA6 | TRA6 | TRA6 |
|  |  | GG2r | GG8r | OPM1 |  |  |  | GG3 | GG13 | GG13 |
|  |  |  | TCA9r | FEM1 |  |  |  |  | GG5r | GG3 |
|  |  |  |  | GG2r |  |  |  |  |  | GG6r |

**Table S3. Reaction deletions of GC strain designs identified by gcOpt and OptKnock for the production of lactate under anaerobic conditions.** Maximal intervention sizes between one and five reaction deletions (1-5 KO) were used to calculate GC strain designs employing the E. coli core model built by Trinh et al. [1]. A fixed growth rate $\mu_{fix}$ of 0.1 h^-1^ was applied for the gcOpt simulations. The reaction deletions of each identified GC strategy are given according to the abbreviations from Trinh et al. [1]. For maximal intervention sizes from three to five OptKnock returned the same deletion strategy.

| gcOpt | | | | |  | OptKnock | | | | |
| --- | --- | --- | --- | --- | --- | --- | --- | --- | --- | --- |
| 1 KO | 2 KO | 3 KO | 4 KO | 5 KO |  | 1 KO | 2 KO | 3 KO | 4 KO | 5 KO |
| - | FEM6 | FC1r | FEM6 | FEM7 |  | FEM6 | FEM6 | FEM6 | FEM6 | FEM6 |
|  | FEM8 | FEM8 | FEM7 | TRA1 |  |  | GG2r | GG2r | GG2r | GG2r |
|  |  | TRA1 | FC1r | FC1r |  |  |  | ANA3 | ANA3 | ANA3 |
|  |  |  | TCA5 | GLB2 |  |  |  |  |  |  |
|  |  |  |  | PPP3 |  |  |  |  |  |  |

| **Table S4. Effect of the relaxation of ATP/ADP and NADH/NAD^+^ conversion as well as the proton translocation on the GCS of the identified GC strain designs for anaerobic conditions**. All values are given in percentage of the total number of identified GC strategies. | | | | | | | | | | |
| --- | --- | --- | --- | --- | --- | --- | --- | --- | --- | --- |
| Anaerobic | | | | | | | | | |  |
|  | sGC | | | | | |  | wGC + hGC | |  |
|  | Total | | No biomass  precursors  accessible | | All biomass  precursors  accessible | |  | Total | |  |
| Relaxation | Abolished GC | No effect | Abolished  GC | No effect | Abolished  GC | No effect |  | Abolished  GC | No effect |  |
| ATP | 47 | 5 | 43 | 6 | 100 | 0 |  | 0 | 89 |  |
| NADH | 50 | 16 | 46 | 17 | 100 | 0 |  | 8 | 89 |  |
| H^+^ | 53 | 0 | 49 | 0 | 100 | 0 |  | 5 | 89 |  |
| ATP+  H^+^ | 61 | 0 | 57 | 0 | 100 | 0 |  | 5 | 89 |  |
| NADH  +H^+^ | 84 | 0 | 83 | 0 | 100 | 0 |  | 11 | 89 |  |
| ATP  +NADH  +H^+^ | 84 | 0 | 83 | 0 | 100 | 0 |  | 11 | 89 |  |

| **Table S5. Effect of the relaxation of ATP/ADP and NADH/NAD^+^ conversion as well as the proton translocation on the GCS of the identified GC strain designs for aerobic conditions.** All values are given in percentage of the total number of identified GC strategies. | | | | | | | | | | |
| --- | --- | --- | --- | --- | --- | --- | --- | --- | --- | --- |
| Aerobic | | | | | | | | | |  |
|  | sGC | | | | | |  | wGC + hGC | |  |
|  | Total | | No biomass  precursors  accessible | | All biomass  precursors  accessible | |  | Total | |  |
| Relaxation | Abolished GC | No effect | Abolished  GC | No effect | Abolished  GC | No effect |  | Abolished  GC | No effect |  |
| ATP | 32 | 7 | 36 | 14 | 31 | 5 |  | 1 | 26 |  |
| NADH | 32 | 20 | 39 | 14 | 30 | 22 |  | 2 | 26 |  |
| H^+^ | 6 | 46 | 14 | 11 | 3 | 57 |  | 2 | 26 |  |
| ATP+  H^+^ | 33 | 7 | 39 | 11 | 31 | 5 |  | 2 | 26 |  |
| NADH  +H^+^ | 35 | 26 | 50 | 0 | 30 | 34 |  | 6 | 26 |  |
| ATP  +NADH  +H^+^ | 52 | 0 | 50 | 0 | 53 | 0 |  | 6 | 26 |  |

**REFERENCES**

1. Trinh CT, Unrean P, Srienc F. Minimal *Escherichia coli* cell for the most efficient production of ethanol from hexoses and pentoses. Appl Environ Microbiol. 2008;74:3634–43. doi:10.1128/AEM.02708-07.

2. Hädicke O, Klamt S. Computing complex metabolic intervention strategies using constrained minimal cut sets. Metab Eng. 2011;13:204–13.
